# The Global Prevalence of Vitamin D Deficiency and Insufficiency in Patients with Multiple Myeloma: A Systematic Review and Meta-Analysis

**DOI:** 10.3390/nu15143227

**Published:** 2023-07-20

**Authors:** Nor Hayati Ismail, Ali Mussa, Mutaz Jamal Al-Khreisat, Shafini Mohamed Yusoff, Azlan Husin, Muhammad Farid Johan, Md Asiful Islam

**Affiliations:** 1 Department of Haematology, School of Medical Sciences, Universiti Sains Malaysia, Kubang Kerian 16150, Malaysia; hayatiismail255@gmail.com (N.H.I.); alimaha1341989@gmail.com (A.M.); mutaz.alkhreisat@student.usm.my (M.J.A.-K.); shafini@usm.my (S.M.Y.); 2 Department of Biology, Faculty of Education, Omdurman Islamic University, Omdurman P.O. Box 382, Sudan; 3 Department of Internal Medicine, School of Medical Sciences, Universiti Sains Malaysia, Kubang Kerian 16150, Malaysia; azlanh@usm.my; 4 WHO Collaborating Centre for Global Women’s Health, Institute of Metabolism and Systems Research, College of Medical and Dental Sciences, University of Birmingham, Birmingham B15 2TT, UK

**Keywords:** multiple myeloma, MM, vitamin D, deficiency, insufficiency, prevalence, meta-analysis

## Abstract

Background: Multiple myeloma (MM) is a hematological malignancy characterized by the exponential growth of malignant plasma cells. Individuals diagnosed with MM exhibit a deficiency in vitamin D and may suffer fatigue, a loss of muscular strength, persistent musculoskeletal aches, and pain. The objective of this systematic review and meta-analysis is to determine the prevalence of vitamin D insufficiency and deficiency in individuals diagnosed with MM. Methods: We searched five electronic databases using relevant keywords. The quality of the included studies was evaluated using the critical appraisal tool developed by the Joanna Briggs Institute. We employed a random-effects model and presented the findings in the form of percentages accompanied by 95% confidence intervals (CI). This protocol has been officially registered in PROSPERO under the registration number CRD42021248710. Results: The meta-analysis comprised a total of eighteen studies and found that, among patients with MM, the occurrence of serum vitamin D deficiency and insufficiency was 39.4% (95% CI: 25.8 to 52.9, n = 3746) and 34.1% (95% CI: 20.9 to 47.2, n = 3559), respectively. The findings indicate that a greater proportion of newly diagnosed patients exhibited vitamin D deficiency and insufficiency, with rates of 43.0% and 41.6%, respectively, compared to those receiving treatment (rates of 41.6% and 32.3%, respectively). The findings of the sensitivity analyses were consistent, and most of the studies (72.2%) were deemed to be of high quality. The results of Egger’s test indicated the absence of publication bias. Conclusions: Patients diagnosed with MM have been found to exhibit significantly elevated levels of both vitamin D deficiency and insufficiency. Therefore, it is recommended to consider vitamin D testing as an additional parameter in the current criteria for the clinical evaluation of MM.

## 1. Introduction

Multiple myeloma (MM) is characterized by the development of neoplastic plasma cells that proliferate clonally within the bone marrow. MM is distinguished by the existence of monoclonal proteins (antibodies) in the blood or urine and is associated with the dysfunction of multiple organs, such as hypercalcemia, renal insufficiency, anemia, and bone destruction, which are collectively known as the CRAB criteria [1]. Plasma cell myeloma (PCM) is considered the second most common form of blood malignancy, surpassed only by non-Hodgkin lymphoma [2]. The prevalence of PCM is observed to be higher in males, especially in their sixth decade of life. Unfortunately, a definitive cure for this condition is yet to be established [3]. The annual incidence of MM is reportedly 4.5–6 cases per 100,000 individuals, with a higher prevalence observed in affluent regions such as North America, Australia, and Western Europe. According to recent research, there has been a significant rise in the global incidence of MM, with a 126.0% increase observed between 1990 and 2016. This trend is attributed to the aging world population, which has increased age-specific incidence [4]. Individuals with comorbidities, genetic predisposition, and exposure to physical toxins, such as pesticides, organic solvents, and radiation, may be at an increased risk of developing MM [5,6]. The utilization of innovative agents, including advanced proteasome inhibitors and immunomodulatory drugs, has substantially enhanced the median five-year survival rate of patients to 82% [7]. Nevertheless, a considerable proportion of new cases have been documented thus far [8]. Most individuals diagnosed with MM encounter relapse frequently, suggesting that this ailment is comparatively resistant to treatment. The most difficult aspect of managing and remedying MM is striking a balance between the effectiveness of myeloma cell eradication and the potential toxicity for patients [9].

The progression of active MM occurs through two pre-malignant stages, namely monoclonal gammopathy of unknown significance (MGUS) and smoldering MM (SMM) [10]. The deregulation of normal plasma cells is initiated by immunoglobulin heavy-chain translocations and hyperdiploidy, which cause multiple genetic mutation events in both stages [11]. The benign condition known as monoclonal gammopathy of undetermined significance (MGUS) has been observed to advance to MM at a yearly rate of 1%. A significant proportion of patients, over 50%, continue to live with the disease for a duration exceeding 10 years before receiving a diagnosis [12]. Additionally, the progression from SMM to MM occurs at a faster rate, with a reported incidence of 10.0% within the first five years post-diagnosis, followed by 3.0% in the subsequent five years, and 1.5% thereafter [13].

Vitamin D is a steroid hormone that is soluble in fat and has been extensively studied for its role in maintaining healthy bones by regulating the balance of calcium and phosphorus. Its importance extends beyond bone health and includes functions such as bone remodeling, cardiovascular health, mineral metabolism, and immunomodulation [14]. The serum 25-hydroxyvitamin D [25(OH)D] assay was utilized to assess the vitamin D status of individuals diagnosed with MM [15]. A cut-off value of ≥30 ng/mL was deemed adequate for this purpose, as per previous studies [16,17]. Vitamin D insufficiency and deficiency are frequently associated with poorer health outcomes, increased malignant cell burden, inadequate response to treatment, and decreased OS and relapse-free survival in hematological cancers and diseases [18]. These outcomes are caused by an increase in apoptosis, reduced angiogenesis, and enhanced cell cycle arrest, resulting in a slower proliferation rate [19,20]. Early signs of alterations in bone microarchitecture and an elevated likelihood of bone fracture are frequently observed in the pre-malignant condition which is known as MGUS [21]. Patients with MGUS experience a 1.4–1.7 times greater occurrence of skeletal fractures [22], with a particular emphasis on vertebral fractures [23]. The deficiency of vitamin D facilitates the advancement of MGUS and SMM to MM by stimulating osteoclasts and enhancing osteoclastogenesis via the generation of various cytokines and molecules that possess the capability of osteoclast-activating factors (OAF) [24], including but not limited to interleukin-6 (IL-6), IL-1β, and IL-3 when interacting with the bone microenvironment [25]. The insufficiency of vitamin D may considerably augment the likelihood of advancement from a premalignant antecedent to MM. It is noteworthy that this inadequacy is often associated with advanced disease states, a heightened susceptibility to bone fractures, and unfavorable prognostic outcomes in MM [26].

Vitamin D has been demonstrated to play a role in immunomodulation, affecting immune system function [27]. Research has demonstrated its ability to modulate diverse immune cells and molecules implicated in immune reactions. In MM, abnormal plasma cells have the ability to evade immune surveillance and inhibit immune responses [28]. Vitamin D deficiency and insufficiency have the potential to modulate the immune function in multiple myeloma (MM) by affecting various immune cells, including T cells, B cells, and natural killer cells [29]. Additionally, vitamin D has been demonstrated to exhibit anti-inflammatory effects by reducing the production of pro-inflammatory cytokines and increasing the production of anti-inflammatory cytokines, leading to the modification of the microenvironment in cases of MM [30]. A number of studies indicate that vitamin D may possess antiproliferative properties against MM cells due to its regulation of cell cycle progression, induction of apoptosis, and inhibition of angiogenesis [24].

Several competitive binding approaches, including HPLC, radioimmunoassay, LC-MS/MS, CLIA, CMIA, ECL, and enzyme immunoassay, have been sanctioned for the evaluation of vitamin D. Prior research has indicated that a vitamin D insufficiency level of 20 ng/mL is commonly linked with unfavorable clinical consequences [31,32]. Additionally, it has been noted that a significant proportion of patients diagnosed with MM exhibit a heightened incidence of vitamin D deficiency. It is worth noting that the evaluation of vitamin D levels is not a routine component of the standard MM diagnostic protocol [33,34,35]. A meta-analysis has evaluated the effect of vitamin D levels on the prognosis of hematological malignancies, including MM, during diagnosis and transplantation [36]. However, a meta-analysis to determine the global prevalence of vitamin D deficiency and insufficiency among MM patients has not been conducted yet, and to the best of our knowledge, there has been no meta-analysis that has estimated the global prevalence of vitamin D deficiency and insufficiency in patients with MM. Therefore, the objective of this systematic review and meta-analysis was to assess the worldwide occurrence of vitamin D insufficiency and deficiency among individuals specifically with MM.

## 2. Materials and Methods

### 2.1. Reporting Guidelines and Protocol Registration

The systematic review and meta-analysis were conducted by the Preferred Reporting Items for Systematic Reviews and Meta-Analyses (PRISMA) [37] and Meta-analysis of Observational Studies in Epidemiology (MOOSE) [38] guidelines. The research methodology, identified by the registration number CRD42021248710 in the PROSPERO database, was recorded in the International Prospective Registry of Systematic Reviews database located at the University of York in the United Kingdom.

### 2.2. Criteria for Eligibility

A comprehensive global search was conducted to identify published studies that have documented the prevalence of vitamin D deficiency and insufficiency in patients diagnosed with MM. A screening process was conducted to identify prospective studies that examined the serum concentrations of vitamin D in adult patients who were diagnosed with MM, with no limitations regarding sex or race, and aged 18 years or older.

### 2.3. Literature Search

The retrieval of studies of interest was conducted based on the eligibility criteria, utilizing five electronic databases, namely PubMed, Scopus, ScienceDirect, Web of Science, and Google Scholar. On 26 April 2022, a search was conducted without language limitations to locate studies about vitamin D deficiency and insufficiency in patients diagnosed with MM. The study utilized a targeted search strategy by employing specific keywords such as myeloma, plasma cell dyscrasias, myelomatosis, myelomatoses, Kahler’s disease, Kahler disease, vitamin D, hypovitaminosis, hydroxyvitamin, and 25 OH D. The search was conducted using a combination of Boolean logical operators (AND and OR) and advanced and expert search options (Table S1). The bibliographic sources of the incorporated investigations were verified to guarantee a comprehensive exploration. The management and elimination of duplicate studies were facilitated by the utilization of EndNote X8 software.

### 2.4. Study Selection

The process of screening for inclusion involved the independent evaluation of the title and abstract, followed by the full text, of all studies obtained via the literature search by two authors (NHI and MJAK). Excluded from consideration were papers of a review nature, case studies, studies involving non-human subjects, as well as private opinions and perspectives. Information sourced from news reports, press releases, blogs, and databases were not deemed acceptable. The issue of inclusion was resolved by a process of deliberation involving MFJ and MAI, ultimately resulting in a consensus.

### 2.5. Data Extraction

The data from the studies included in the analysis were independently evaluated by two authors, namely NHI and MAI. Before data extraction, studies composed in a language other than English were subjected to translation into English via the utilization of Google Translate. The information obtained from the studies that were incorporated was transcribed onto a pre-established spreadsheet using Excel. The dataset comprised various parameters, such as the nature of the study, the geographical location of the country, the latitude of the location, the number of patients diagnosed with MM, the stage of MM, the type of patients afflicted with MM, the age of the participants, the method used for measuring vitamin D, the threshold for vitamin D insufficiency and deficiency, the current treatment status, and the parental status regarding vitamin D supplementation. The authors engaged in a discourse to establish a collective agreement in situations in which data were incongruous, equivocal, or absent. In the event of persistent issues, the corresponding or primary author of the relevant studies was contacted via email to request clarification.

### 2.6. Quality Assessment and Publication Bias

The quality of the studies included in the research was evaluated using the critical appraisal tools provided by the Joanna Briggs Institute. The categorization of studies into poor quality (high risk of bias), moderate quality (moderate risk of bias), or high quality (low risk of bias) was based on the total scores falling within the ranges of ≤49%, 50–69%, or ≥70%, as per sources [39,40]. A funnel plot was generated to assess the presence of publication bias via the evaluation of the estimated prevalence of the standard error. Egger’s test was employed to verify the asymmetry of the funnel plot.

### 2.7. Data Analyses

The units of measurement for serum vitamin D levels were standardized to ng/mL. This study reports the median difference (MD) and 95% confidence interval (CI) for the data of the vitamin D levels. A statistical significance level of *p* < 0.05 was used to determine significance. A subgroup analysis was conducted on the vitamin D levels, stratified by patient type and geographical location. The study conducted a tau-squared test to examine heterogeneity (I2) and evaluate inconsistency among the studies that were included. The statistical significance level was set at *p* < 0.05. When the I2 value is close to zero, it suggests that the homogeneity is better. Specifically, an I2 value falling within the range of 25–50% indicates low heterogeneity, while an I2 value between 51–75% suggests moderate heterogeneity. On the other hand, an I2 value exceeding 75% indicates substantial heterogeneity. The quality of each study included in the research was assessed by two authors (NHI and MJAK) using critical appraisal tools. Furthermore, the study employed sensitivity analyses and Galbraith plots to assess the robustness of the findings and identify potential sources of heterogeneity. The study conducted sensitivity analyses wherein small studies, as well as low- and moderate-quality studies, were excluded. Moreover, only cross-sectional studies were considered to estimate the prevalence of vitamin D deficiency and insufficiency in patients diagnosed with MM. The statistical analyses and graphical representations were conducted using RevMan software (version 5.3.5) and RStudio (version 1.1.463) with the metafor package (version 2.0-0) of the R software (version 3.5.1).

## 3. Results

### 3.1. Selection and Inclusion of Studies

**Figure 1:**
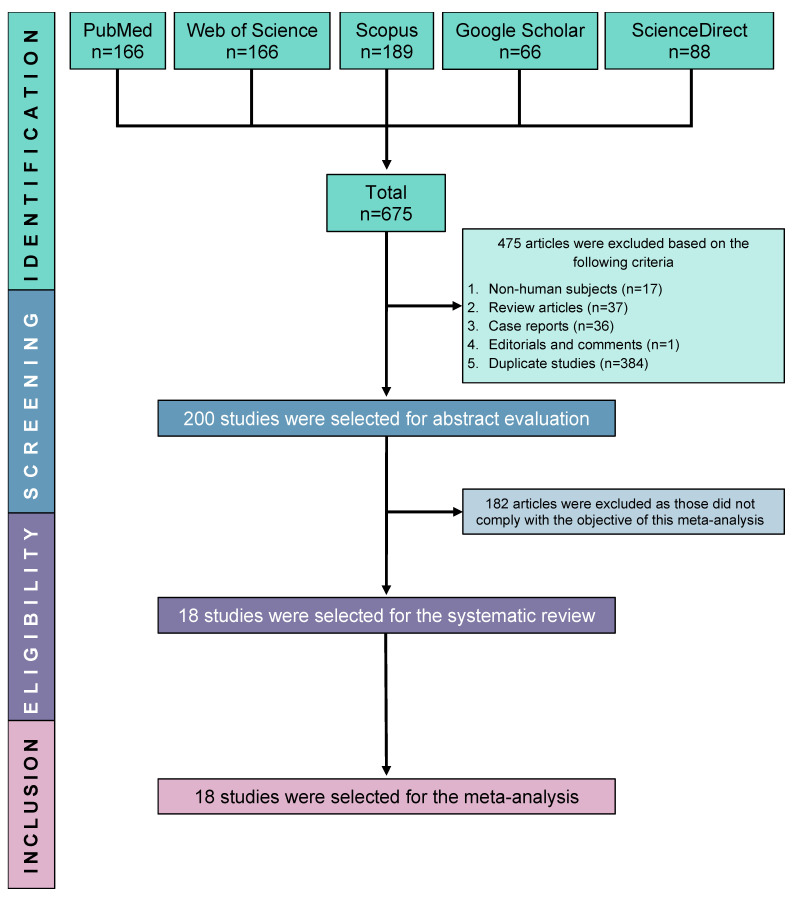
PRISMA flow diagram of study selection.

The results of the database search revealed that 675 studies fulfilled the initial screening criteria. However, 475 studies were excluded from further analysis due to their classification as duplicate studies (n = 384), review articles (n = 37), case reports (n = 36), or non-human studies (n = 17). A comprehensive evaluation of 200 studies was conducted by a meticulous scrutiny of their titles, abstracts, and complete texts to determine their eligibility. After a thorough screening process, a total of 18 studies were deemed suitable for incorporation in this systematic review and subsequent meta-analysis, as depicted in the PRISMA flow diagram (Figure 1).

### 3.2. Study Characteristics

Table 1 presents a summary of the primary features of the incorporated studies [26,33,41,42,43,44,45,46,47,48,49,50,51,52,53,54,55,56]. The studies incorporated in the analysis were carried out across various continents, including North America (n = 6; USA), Europe (n = 7; United Kingdom, France, Switzerland, the Netherlands, Bulgaria, and Turkey), Australia (n = 2), Asia (n = 2; Philippines and South Korea), and Africa (n = 1; Egypt). The study participants’ mean age spanned from 56.0 to 69.1 years and included various categories of patients with MM. Specifically, seven were under treatment (UT), six were NDMM but had not yet commenced treatment, four were NDMM and UT, and one study did not provide information on patient type. Various methodologies have been employed to assess the levels of vitamin D, encompassing both single and combined chemiluminescent immunoassay [42,47,48], ELISA [43,56], tandem mass spectrometry [49], liquid chromatography–tandem mass spectrometry [50], liquid chromatography–tandem mass spectrometry or high-performance liquid chromatography [26], and liquid chromatography–tandem mass spectrometry or immunoassay [51]. The methodology for measuring vitamin D levels was not defined in nine studies [33,41,44,45,46,52,53,54,55]. Different cut-off values (expressed in ng/mL) were utilized to ascertain adequate, inadequate, and deficient serum vitamin D concentrations in individuals diagnosed with MM. Out of the eighteen studies that were included, seven of them reported that the patients were UT. Four studies have documented the consumption of vitamin D supplements among individuals diagnosed with MM [41,48,50,52].

### 3.3. Main Results: Vitamin D Levels in MM

**Figure 2:**
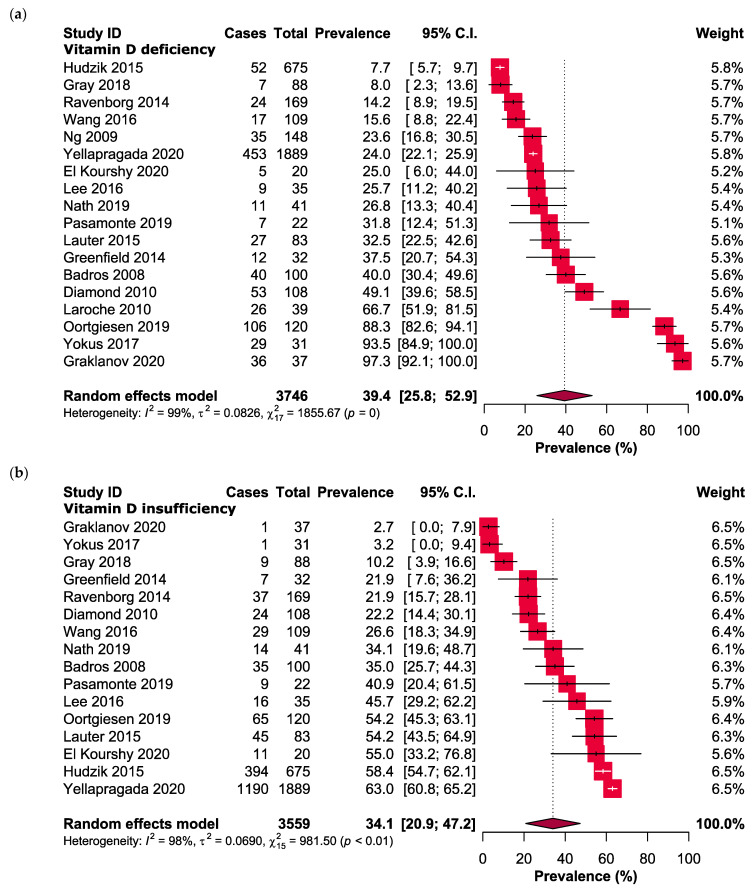
Forest plots presenting the prevalence of (**a**) vitamin D deficiency and (**b**) vitamin D insufficiency in MM patients.

The study findings indicate that the occurrence of vitamin D insufficiency and deficiency was estimated to be 34.1% (95% CI: 20.9–47.2, n = 3559) and 39.4% (95% CI: 25.8–52.9, n = 3746), respectively, as depicted in Figure 2.

### 3.4. Subgroup Analyses

In the cohort of NDMM and treated patients, it was found that 43.0% (95% CI: 6.8 to 79.1) and 41.6% (95% CI: 19.3 to 64.0) exhibited vitamin D deficiency, while 30.2% (95% CI: 3.2 to 57.2) and 32.3% (95% CI: 10.0 to 54.5) had vitamin D insufficiency, as presented in Table 2 and Figure S1. The prevalence of vitamin D deficiency among patients with MM was found to vary based on geographical location. The highest prevalence was observed in Europe, with a rate of 60.0% (95% CI: 24.9 to 91.9), followed by Australia (30.1%; 95% CI: 22.8 to 37.5), Asia (27.9%; 95% CI: 16.3 to 39.5), Africa (25.0%; 95% CI: 6.0 to 44.0), and North America (20.4%; 95% CI: 11.8 to 28.9). Despite variations across different regions, the prevalence of vitamin D insufficiency was found to be the lowest in Europe (24.1%; 95% CI: 6.4 to 41.8), followed by Australia (26.6%; 95% CI: 15.3 to 37.8), North America (41.3%; 95% CI: 25.5 to 57.0), Asia (43.8%; 95% CI: 31.0 to 56.7), and Africa (55.0%; 95% CI: 33.2 to 76.8) among patients with MM.

### 3.5. Quality Assessment

**Figure 3:**
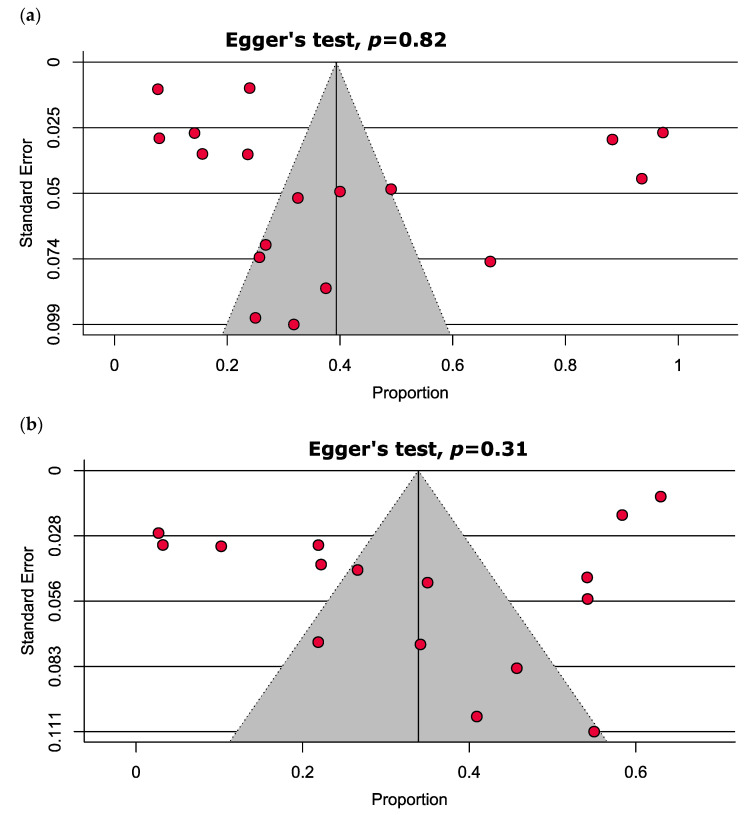
Funnel plots estimating the prevalence of (**a**) vitamin D deficiency and (**b**) vitamin D insufficiency in MM patients depict no significant publication bias.

The comprehensive presentation of the evaluation of the studies that were incorporated is delineated in Tables S2–S4. To summarize, the studies that were analyzed in this study were categorized as high quality (72.2%), moderate quality (22.2%), or low quality (5.6%), as indicated in Tables S2–S4. The findings from the funnel plot and Egger’s test indicate a lack of publication bias in the estimation of vitamin D deficiency (*p* = 0.82) and insufficiency (*p* = 0.31), as illustrated in Figure 3.

### 3.6. Heterogeneity and Sensitivity Analysis

The results of sensitivity analyses indicate that the highest prevalence of vitamin D deficiency was observed when only cross-sectional studies were considered, with a rate of 45.2% (95% CI: 5.8 to 84.5). This was followed by excluding studies of low and moderate quality, which resulted in a prevalence of 41.0% (95% CI: 25.0 to 57.0), and excluding small studies, which resulted in a prevalence of 32.7% (95% CI: 17.8 to 47.6). These findings are presented in Table 3 and Figure S2. Table 3 and Figure S2 demonstrate that the prevalence of vitamin D insufficiency varies depending on the exclusion criteria applied. Excluding small studies resulted in the highest prevalence of 40.4% (95% CI: 26.6 to 54.2), followed by excluding low- and moderate-quality studies with a prevalence of 38.1% (95% CI: 22.7 to 53.4). Considering only cross-sectional studies resulted in a prevalence of 33.8% (95% CI: 7.2 to 60.5) for vitamin D insufficiency.

**Figure 4:**
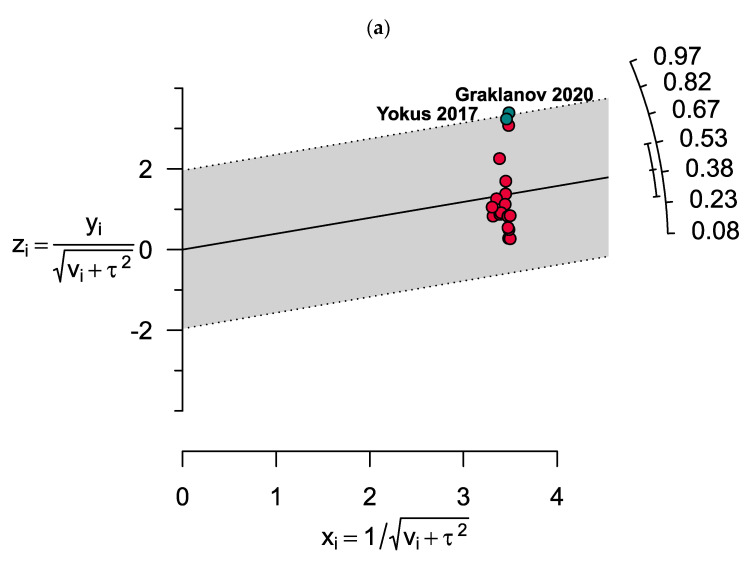

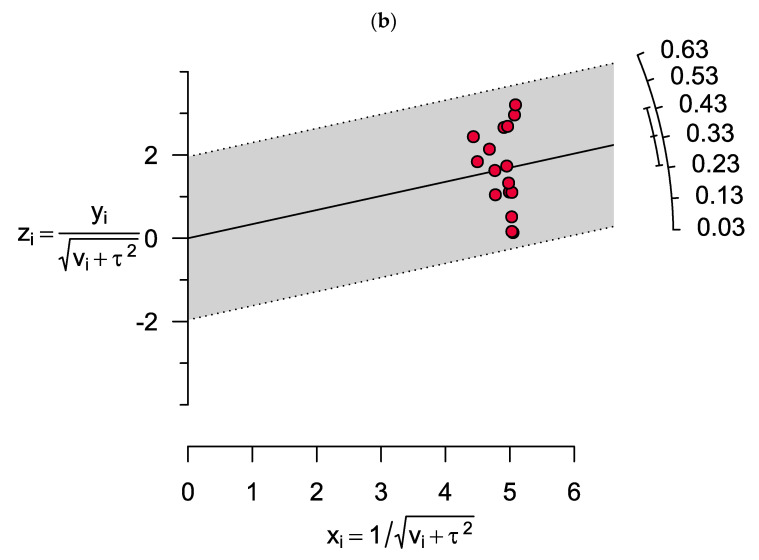
Galbraith plots show two outlier studies in estimating the prevalence of (**a**) vitamin D deficiency. However, there were no outlier studies when assessing (**b**) vitamin D insufficiency in MM patients.

The results showed significant heterogeneity for both vitamin D deficiency and insufficiency among MM patients (I2 = 99%, *p* < 0.0001 and I2 = 98%, *p* < 0.0001, respectively). Two outlier studies in estimating the prevalence of vitamin D deficiency were determined as illusory in the Galbraith plot (Figure 4). However, no outlier study was found when assessing vitamin D insufficiency in MM patients.

## 4. Discussion

Our study found that 34.1% and 39.4% of MM patients were found to have vitamin D deficiency and insufficiency, respectively. Previous studies have also shown that prevalence in both vitamin D deficiency and insufficiency are associated with unfavorable prognoses [34,57]. Vitamin D insufficiency has been reported as a predictor for poor OS among MM patients, even after controlling for age and stage [52]. Oortgiesen et al. and Yokus et al. also revealed a significant proportion of MM cases attributed to the deficiency of vitamin D among patients with MM [53,54].

To the best of our knowledge, this is the first conducted systematic review and meta-analysis assessing global vitamin D insufficiency and deficiency in MM patients. The subgroup analysis showed that both NDMM patients (43.0%) and those receiving insufficient therapy (32.3%) had a higher prevalence of vitamin D deficiency and insufficiency. The results of our meta-analysis are in line with earlier data from the National Health and Nutrition Examination Survey (NHANES), which covered the years 2000 to 2004 and revealed that 30% of respondents aged 50 and over had 25(OH)D levels below the average value [58]. However, another study found that only 24% of NDMM patients had vitamin D levels that were <20 ng/mL [26].

This study demonstrated that slightly higher vitamin D deficiency levels were reported in the NDMM and undertreated patients (43.0%) compared to treated patients (41.6%). However, the level of vitamin D insufficiency was found higher in UT patients compared to NDMM patients at 32.3% and 30.2%, respectively. Additionally, a previous study reported that the portion of MM patients with vitamin D deficiency (40%) reflected serum vitamin D levels below 36 nmol/L. Furthermore, it has been seen that NDMM patients show a lower level of vitamin D in comparison to those who are UT. This finding has been suggested as a surrogate indicator for assessing the clinical condition of MM patients [56]. The prevalence of vitamin D insufficiency tends to increase during the latter stages of MM, and this has been observed to have a negative impact on the OS of patients [48,53]. A study conducted earlier revealed that individuals afflicted with metastatic bone disease and MM exhibited a significantly elevated incidence of vitamin D insufficiency [59]. Moreover, NDMM patients who exhibited a vitamin D deficiency status below 50 nmol/L (20 ng/mL) demonstrated increased mean values for serum C-reactive protein (CRP) and creatinine, as well as decreased serum albumin levels. Furthermore, there was a positive correlation between vitamin D insufficiency and the International Staging System (ISS), suggesting that the presence of vitamin D deficiency could potentially serve as an indicator of unfavorable prognostic outcomes in patients with MM [26].

The prevalence of vitamin D insufficiency or deficiency is extensive across various age groups of patients and is primarily attributed to the insufficient intake of vitamin D and/or limited exposure to sunlight [60]. Moreover, it has been suggested that vitamin D may have a potential role in both myeloma bone disease and MM [61]. Other factors including geographical variables, such as latitude and solar irradiance, have an impact on the vitamin D levels of the general populace [62]. In general, countries near the equator experience a higher incidence of solar radiation throughout the year in contrast to those situated farther away from the equator. Nonetheless, the prevalence of sun-seeking conduct is infrequent among these communities owing to the prevalent hot climate [63]. In addition, previous research indicates that individuals residing at greater distances from the equator tend to exhibit lighter skin pigmentation [64]. For example, another study has indicated a correlation between individuals with darker skin typology and reduced DNA damage, suggesting a potential protective effect against skin cancer. Our results found that the prevalence of vitamin D deficiency among patients with MM was found to vary based on geographical location. The highest prevalence of vitamin D deficiency was observed in Europe (60.0%), Australia (30.1%), Asia (27.9%), Africa (25.0%), and North America (20.4%). However, the prevalence of vitamin D insufficiency was found to be the lowest in Europe (24.1%), Australia (26.6%), North America (41.3%), Asia (43.8%), and Africa (55.0%) among patients with MM. The interplay between vitamin D and racial disparities in its physiological effects is a subject of ongoing research, with differing viewpoints. Nevertheless, a study found that African American (AA) patients had a significantly higher rate of vitamin D deficiency (82.1%) compared to white patients (30.9%) [65]. In white patients, vitamin D deficiency was associated with significantly lower OS compared to patients with normal vitamin D levels, as supported by the spike hazard of mortality by 38%. Conversely, there was no observed difference in the OS between African American (AA) patients with vitamin D deficiency and those with normal levels of serum vitamin D [52]. Vitamin D is essential for various physiological processes, such as immune function, bone health, and potentially tumor suppression. Evidence suggests potential racial and ethnic variations in the response to vitamin D levels and their physiological impacts. Additional factors, including genetic variations, cultural practices, socioeconomic factors, and geographic location, can also affect vitamin D levels and their physiological effects. Hence, it is crucial to take into account various factors when investigating the relationship between vitamin D, racial disparities, and their potential impact on tumor suppression or other health outcomes.

The synthesis of vitamin D in the skin also decreases with advancing age, which is a common occurrence among individuals diagnosed with multiple myeloma [66,67]. The process of aging is linked to a reduction in the concentration of 7-DHC in the skin, which, in one study, led to a decrease of over four times in the production of vitamin D3 in a 70-year-old individual compared to a 20-year-old adult [66,67]. Furthermore, the elderly population tends to remain indoors for extended periods and exhibit restricted physical activity as a result of multiple co-morbidities, thereby exacerbating their reduced exposure to sunlight [68].

The clinical significance of vitamin D and its metabolites in patients with MM is attributed to their participation in the regulation of calcium homeostasis and bone metabolism [24]. Vitamin D deficiency and insufficiency have been observed to cause a direct and erosive degradation of bone tissues, which is characterized by the manifestation of typical osteoclast markers, such as the formation of osteoclasts [69,70]. The insufficiency of vitamin D triggers the activation of TNF-related activation-induced cytokines (TRANCE), osteoprotegerin (OPG) pathways, and various intracellular signaling pathways such as receptor activator of nuclear factor kappa-B ligand (RANKL)–receptor activator of NF-κB (RANK)–OPG RANK-RANKL-OPG, NOTCH, and Wnt. This leads to the degradation of bone by osteoclasts and decreased bone mineralization [71]. Alterations in bone metabolism resulting from the overexpression of RANKL and a decrease in the OPG/RANKL ratio have been observed in the asymptomatic phase of MGUS and SMM. During this phase, patients are at a higher risk of developing osteoporosis, which can result in reduced bone strength and modified bone morphology due to the influence of inhibitory factors [72,73,74]. Alterations in bone metabolism resulting from the overexpression of RANKL and the decoy receptor OPG have been observed to impact osteoblasts and osteoclastogenesis, potentially contributing to the pathogenesis of certain diseases [75,76,77]. A recent investigation has demonstrated a connection between the accelerated modification of bone metabolism by RANKL and OPG and an elevated likelihood of transitioning from MGUS to MM [54]. Furthermore, other bone resorption markers and bone formation markers have demonstrated the potential to provide valuable clinical insights in a broad myeloma population and aid in decision making during routine clinical practice. Recently, Bao et al. investigated the influence of the serum ratio of vitamin D to carboxy-terminal telopeptide of type I collagen (β-CTX) on progression-free survival (PFS) and OS (OS) in NDMM patients. The study findings indicated a negative correlation between serum vitamin D levels and β-CTX. The group with a lower vitamin D-to-β-CTX ratio demonstrated hypocholesterolemia and inferior PFS and OS, as well as a higher prevalence of ISS stage-III and R-ISS stage-III, increased plasma cells in the BM, and elevated serum calcium levels compared to the group with a higher vitamin D-to-β-CTX ratio. The vitamin D-to-β-CTX ratio is a significant biomarker for identifying high-risk cases with poor prognosis in NDMM patients. Remarkably, β-CTX outperforms vitamin D alone for forecasting progression-free survival (PFS) and OS (OS) in NDMM [78].

A previous study found that most patients with multiple myeloma (MM) exhibit vitamin D deficiency, but this deficiency does not appear to be linked to high-risk cytogenetics [46]. One case report, however, discovered that vitamin D deficiency was demonstrated in a 33-year-old man with MM who possessed four poor prognostic features, including the amplification of 1q21, the translocation of t(4;14), the deletion of 6q21 and 13q14, and a decreased chromosome count to 44, X,-Y [79].

Another factor leading to vitamin D deficiency and insufficiency is the role of the intranuclear VDR, which mediates the developmental regulatory effects of vitamin D [1,25(OH)2D3] [80]. The VDR gene has been identified as a potential biomarker for evaluating the risk of patients with MM as it can modify clinical symptoms of bone diseases, including bone mass [81]. A study of Kashmiri populations has demonstrated that the FokI polymorphism, characterized by the ff genotype, is linked to a heightened vulnerability to the onset and advancement of MM [82]. According to a study conducted in Fujian Province, China, there is a significant correlation between the elevated frequencies of the A allele in the BsmI or the C allele in the TaqI polymorphisms and an augmented susceptibility to MM [81]. A previous investigation indicated a positive correlation between the T allele of the FokI gene located within the VDR gene and an elevated susceptibility to MM [83]. Several additional studies have reported that VDR polymorphism has an impact on the occurrence of vitamin D deficiency in MM patients [84,85,86]. This variable may potentially contribute to the progression of the disease. Moreover, the evaluation of vitamin D is a feasible approach for addressing the resistance to immunomodulatory therapy [87,88,89,90]. For example, the transcription of the anti-inflammatory dual-specificity protein phosphatase 1 (DUSP1) gene is upregulated by vitamin D, while the production of the inflammatory chemokine IL-8 is downregulated by vitamin D when it binds to its receptors. This process is mediated by hyperinflammatory macrophages [89].

The definition of vitamin D deficiency and insufficiency has been a topic of ongoing discussion as the threshold for vitamin D levels has varied across different studies. Our study adhered to the current consensus that the prevailing standard for vitamin D deficiency is a serum level of up to 20 ng/mL, while levels between 20 and 29 ng/mL are considered insufficient, and levels of ≥30 ng/mL are considered sufficient [91]. There was a correlation reported between inadequate levels of vitamin D, specifically 20 mg/mL serum 25-hydroxyvitamin D [25(OH)D], and compromised skeletal health, including osteoporosis [92]. Therefore, it is recommended to initiate treatment when the 25(OH)D level is at 20 ng/mL [24,93]. In addition, it has been observed that individuals with a 25(OH)D level of less than 12 ng/mL, which is considered a severe level of vitamin D deficiency, are at a higher risk of contracting infections and suffering from various other disorders [94]. Furthermore, this condition may also lead to an increased mortality rate in MM patients [95,96]. A study evaluating the prevalence of vitamin D deficiency recognized that MM patients with serum vitamin D levels below 10 ng/mL had a higher presence of plasma cells in their bone marrow (44.8% vs. 13.3%). Following supplementation, vitamin D levels in groups with vitamin D deficiency and insufficiency without renal insufficiency significantly improved; nevertheless, these groups still did not reach acceptable levels. To attain acceptable long-term 25(OH)D levels during follow-up, patients with MM must take greater doses of vitamin D as supplements. After supplementation, the vitamin D level significantly rose in hemoglobin (11.8 to 12.3; *p* = 0.039), leukocytes (4.9 to 5.8; *p* = 0.011), and erythrocytes (3.8 to 4.0; *p* = 0.004); however, it significantly reduced in thrombocytes (200.5 to 175.2; *p* = 0.036) [48]. The use of bisphosphonates, such as zoledronic acid or pamidronic acid, as supplements, was once the gold standard for treating MM patients to avoid the negative effects of bone disease [97,98] because their binding to the exposed area of hydroxyapatite crystals makes the process of bone remodeling easier [99,100]. By blocking intracellular farnesyl pyrophosphate synthase, this pyrophosphate analog inhibits osteoclast endocytosis, reducing osteoclast death and bone loss [101]. The most recent policy review of the International Myeloma Working Group’s Bone Working Group recommends zoledronic acid as a bone-targeted drug for NDMM with or without MM-related bone damage. A decrease in dosage frequency or the termination of supplementation after patients have received monthly zoledronic acid for at least 12 months should be regarded as a sign of a very excellent or better partial response [102]. By limiting the binding of RANKL to RANK and consequently reducing bone resorption, denosumab, a different clinically produced targeted drug, functions as a monoclonal antibody against RANKL that replicates the physiological action of OPG [103]. In NDMM patients with concomitant bone disease and autologous stem cell transplant candidates, denosumab may prolong PFS. Denosumab is often preferable to zoledronic acid for delaying the start of the first skeletal-related event after a diagnosis of MM [104] and for lowering renal toxicity, especially in individuals with renal impairment and refractoriness to zoledronic acid [105,106,107].

Performing this systematic review and meta-analysis has shown many potential advantages. The incidence of vitamin D deficiency and insufficiency among patients with MM is being reported in this systematic review and meta-analysis for the first time. This research was built on a thorough review of the literature using five reputable internet databases with no time or language constraints. The findings, which represent worldwide outcomes by spanning five continents (i.e., North America, Europe, Africa, Australia, and Asia), have been described using significant sample sizes: n = 3754 and 3559 for vitamin D deficiency and insufficiency, respectively. To determine how publication bias influenced each of the listed papers, the funnel plot and Egger’s test were used. Furthermore, this meta-analysis study’s conclusion is solidly supported by similar statistical findings after further sensitivity testing. In our meta-analysis study, 72.2% of the papers were of good quality. However, several restrictions may be considered. First off, there were just eighteen included papers in this meta-analysis, which is a rather small amount. Second, there was significant variation in the analyses of vitamin D sufficiency and shortage in MM patients.

## 5. Conclusions

In conclusion, our meta-analysis showed that there was a substantially higher incidence of vitamin D insufficiency (34.1%) and deficiency (39.4%) among MM patients. Patients who had just been diagnosed and those who were receiving therapy were more likely to suffer from vitamin D deficiency and insufficiency, especially those residing in Europe and Africa. To maintain optimal blood levels of vitamin D and stop the course of the disease, vitamin D supplementation should be thought of as a part of MM health care procedures. Given the fact that patients with MM often have vitamin D insufficiency, vitamin D testing needs to be taken into consideration as a diagnostic requirement and a regular parameter to track the disease’s progression.

## Figures and Tables

**Table 1:** Major characteristics of the included studies.

No.	Study ID [References]	Type of Study	Country, Location (Latitude)	Number of MM Patients (Female%), MM Stage (%)	Types of MM Patients	Age of the Participants (Mean ± SD/Median (IQR)/Range) (Years)	Vitamin D Measurement Method	Cut-Off for Vitamin D Insufficiency; Deficiency (ng/mL)	Current Treatment Status	Were the Patients on Vitamin D Supplements?
1	Badros 2008 [41]	Cohort	USA, Baltimore, 39.2904° N	100 (42.0), NR	NDMM	59.0 (29.0–80.0)	NR	14–30; ≤14	None	Yes, 20%
2	Diamond 2010 [42]	Cohort	Australia, New South Wales, 31.2532° S	108 (46.0), NR	UT	69.1 ± 10.5	Chemiluminescent immunoassay	20–30; <20	NR	NR
3	El Kourshy 2020 [56]	Case-control	Egypt, Cairo, 30.0444° N	60 (NR), NR	NDMM and UT	NR	ELISA	10–30; <10	NR	NR
4	Graklanov 2020 [43]	Cross-sectional	Bulgaria, Plovdiv, 42.1354° N	37 (51.4), Stage I: 13.5 Stage II: 13.5 Stage III: 70.0	NDMM	68.0 ± NR	ELISA	20–30; <20	None	NR
5	Gray 2018 [44]	Cohort	Gloucester, United Kingdom 51.8642° N	88 (NR), NR	NDMM	NR	NR	NR	None	NR
6	Greenfield 2014 [45]	Cross-sectional	Sheffield, United Kingdom, 53.3811° N	32 (47.0), NR	UT	61.0 (41.0–71.0)	NR	12–20; <12	Cyclophosphamide, melphalan, high-dose steroids, doxorubicin, vincristine, thalidomide, bortezomib, lenalidomide, fludarabine, etoposide, cytarabine, cisplatin, and/or interferon alpha	NR
7	Hudzik 2015 [46]	Cross-sectional	Ohio State, USA, 40.4173° N	675 (NR), Stage I: 28.6 Stage II: 48.7 Stage III: 21.9	NDMM	64.0 (28.0–95.0)	NR	10–30; <10	NR	NR
8	Laroche 2010 [47]	Cohort	Toulouse, France, 43.6047° N	39 (59.0), NR	NDMM and UT	56.0 ± 6.6	Chemiluminescent immunoassay	<20; NR	High-dose chemotherapy	NR
9	Lauter 2015 [48]	Cohort	Bonn, Germany, 50.7374° N	83 (38.5), NR	NDMM and UT	66.3 (43.0–86.0)	Chemiluminescence immunoassay	10–30; <10	NR	Yes
10	Lee 2016 [49]	Cross-sectional	Bucheon, South Korea, 37.5034° N	35 (42.9), NR	NDMM	64.0 (60.0–74.0)	Tandem mass spectrometry	10–20; <10	Analgesic, acetaminophen, and opioid	NR
11	Nath 2019 [50]	Cross-sectional	Townsville, Australia, 19.2590° S	41 (76.0), Stage I: 47.0 Stage II: 42.0 Stage III: 11.0	UT	69.0 (45.0–90.0)	Liquid chromatography–tandem mass spectrometry	8–12; <20	Bisphosphonate, alkylators, steroids, proteasome inhibitors, immunomodulatory agents, monoclonal antibodies, and/or bisphosphonates	Yes
12	Ng 2009 [26]	Cohort	Rochester, USA, 43.1566° N	148 (38.0), NR	NDMM	60.3 (56.6–63.9)	Liquid chromatography–tandem mass spectrometry or high-performance liquid chromatography	NR; <20	None	NR
13	Oortgiesen 2019 [54]	Cross-sectional	Leeuwarden, Netherlands, 53.2012° N	120 (42.5), NR	UT	68.0 (48.0–84.0)	NR	NR; <30	NR	NR
14	Pasamonte 2019 [55]	Cross-sectional	Manila, Philippines, 14.5995° N	22 (55.0), NR	NDMM and UT	61.3 ± 10.0	NR	21–29; <20	NR	NR
15	Ravenborg 2014 [33]	Cohort	West Hollywood, USA, 34.0900° N	169 (38.0), NR	NR	65.4 (38.0–85.0)	NR	20–30; <20	NR	NR
16	Wang 2016 [51]	Cohort	California, USA, 36.7783° N	111 (46.0), NR	UT	66.0 (42.0–89.0)	Liquid chromatography–tandem mass spectrometry or immunoassay	20.0–29.9; <20	Bortezomib and/or thalidomide	NR
17	Yellapragada 2020 [52]	Cohort	Boston, USA, 42.3601° N	1889 (3.2), Stage I: 18.3 Stage II: 30.2 Stage III: 51.5	UT	68.9 ± 10.2	NR	NR; <20	NR	Yes
18	Yokus 2017 [53]	Cohort	Istanbul, Turkey, 41.0082° N	30 (36.7), Stage I: 16.1 Stage II: 35.5 Stage III: 48.4	UT	63.0 (49.0–90.0)	NR	21–29; ≤20	NR	NR

MM: Multiple myeloma; NR: not reported; NDMM: newly diagnosed MM; UT: under treatment; ELISA: enzyme-linked immunosorbent assay; IMWG: international myeloma working group.

**Table 2:** Subgroup analyses.

Strategies of Subgroup Analyses	Prevalence [95% CIs] (%)	Number of Studies Analyzed	Total Number of Subjects	Heterogeneity	
				*I* ^2^	*p*-Value
**Vitamin D deficiency**					
NDMM	43.0 [6.8–79.1]	6	385	99%	<0.0001
UT	41.6 [19.3–64.0]	7	2488	99%	<0.0001
Europe	60.7 [29.4–91.9]	7	430	99%	<0.0001
North America	20.4 [11.8–28.9]	6	3090	97%	<0.0001
Australia	30.1 [22.8–37.5]	2	149	0%	0.57
Asia	27.9 [16.3–39.5]	2	57	0%	0.62
Africa	25.0 [6.0–44.0]	1	20	NA	NA
**Vitamin D insufficiency**					
NDMM	30.2 [3.2–57.2]	5	935	99%	<0.0001
UT	32.3 [10.0–54.5]	7	2330	99%	<0.0001
Europe	24.1 [6.4–41.8]	6	391	97%	<0.0001
North America	41.3 [25.5–57.0]	5	2942	98%	<0.0001
Australia	26.6 [15.3–37.8]	2	149	50%	0.15
Asia	43.8 [31.0–56.7]	2	57	0%	0.72
Africa	55.0 [33.2–76.8]	1	20	NA	NA

CIs: Confidence intervals; NA: not applicable; NDMM: newly diagnosed MM; UT: under treatment.

**Table 3:** Sensitivity analyses.

Strategies of Sensitivity Analyses	Prevalence [95% CIs] (%)	The Difference in Pooled Prevalence Compared to the Main Result	Number of Studies Analyzed	Total Number of Subjects	Heterogeneity	
					*I^2^*	*p*-Value
**Vitamin D deficiency**						
Excluding small studies	32.7 [17.8–47.6]	6.7% lower	8	3318	99%	<0.0001
Excluding low- and moderate-quality studies	41.0 [25.0–57.0]	1.6% higher	14	3418	99%	<0.0001
Considering only cross-sectional studies	45.2 [5.8–84.5]	5.8% higher	7	962	100%	<0.0001
**Vitamin D insufficiency**						
Excluding small studies	40.4 [26.6–54.2]	6.3% higher	7	3170	98%	<0.0001
Excluding low- and moderate-quality studies	38.1 [22.7–53.4]	4.0% higher	12	3231	99%	<0.0001
Considering only cross-sectional studies	33.8 [7.2–60.5]	0.3% lower	6	842	98%	<0.0001

CIs: Confidence intervals.

## Data Availability

The data presented in this study are available in the main text and Supplementary Materials.

## Supplementary Materials

The following supporting information can be downloaded at https://www.mdpi.com/article/10.3390/nu15143227/s1. Figure S1: Subgroup analyses estimating the prevalence of vitamin D deficiency and insufficiency in (A,B) patients with NDMM, (C,D) patients under treatment, and (E–N) MM patients from different regions; Figure S2: Sensitivity analyses (A,B) excluding small studies, (C,D) excluding low- and moderate-quality studies, and (E,F) considering only cross-sectional studies estimating the prevalence of vitamin D deficiency and insufficiency in MM patients; Table S1: Search strategies; Table S2: Quality assessment of the included cohort studies; Table S3: Quality assessment of the included cross-sectional studies; Table S4: Quality assessment of the included case-control studies.
