# Supplementary material for: The Global Prevalence of Vitamin D Deficiency and Insufficiency in Patients with Multiple Myeloma: A Systematic Review and Meta-Analysis
Source: Nutrients. 2023 Jul 20;15(14):3227. doi: 10.3390/nu15143227 (PMC10386623; doi:10.3390/nu15143227)
Supplement: Supplementary file 1 [file nutrients-15-03227-s001.zip › nutrients-2500131-supplementary.pdf]

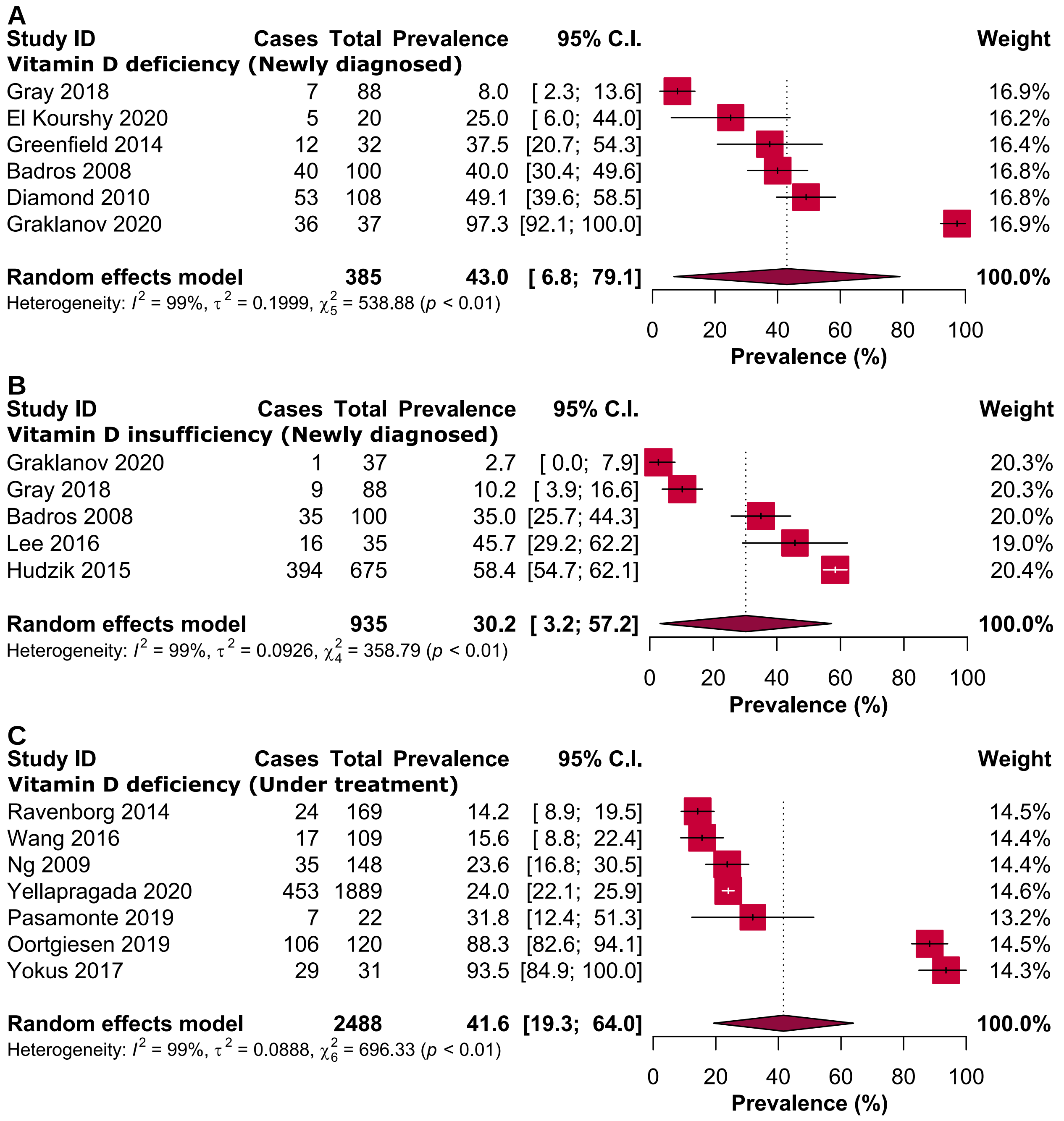
A
Study ID Cases Total Prevalence 95% C.I. Weight
Vitamin D deficiency (Newly diagnosed)
Gray 2018 7 88 8.0 [2.3; 13.6] 16.9%
El Kourshy 2020 5 20 25.0 [6.0; 44.0] 16.2%
Greenfield 2014 12 32 37.5 [20.7; 54.3] 16.4%
Badros 2008 40 100 40.0 [30.4; 49.6] 16.8%
Diamond 2010 53 108 49.1 [39.6; 58.5] 16.8%
Graklanov 2020 36 37 97.3 [92.1; 100.0] 16.9%
Random effects model 385 43.0 [6.8; 79.1] 100.0%
Heterogeneity: I^2 = 99%, τ^2 = 0.1999, χ^2_5 = 538.88 (p < 0.01)
0 20 40 60 80 100
Prevalence (%)
B
Study ID Cases Total Prevalence 95% C.I. Weight
Vitamin D insufficiency (Newly diagnosed)
Graklanov 2020 1 37 2.7 [0.0; 7.9] 20.3%
Gray 2018 9 88 10.2 [3.9; 16.6] 20.3%
Badros 2008 35 100 35.0 [25.7; 44.3] 20.0%
Lee 2016 16 35 45.7 [29.2; 62.2] 19.0%
Hudzik 2015 394 675 58.4 [54.7; 62.1] 20.4%
Random effects model 935 30.2 [3.2; 57.2] 100.0%
Heterogeneity: I^2 = 99%, τ^2 = 0.0926, χ^2_4 = 358.79 (p < 0.01)
0 20 40 60 80 100
Prevalence (%)
C
Study ID Cases Total Prevalence 95% C.I. Weight
Vitamin D deficiency (Under treatment)
Ravenborg 2014 24 169 14.2 [8.9; 19.5] 14.5%
Wang 2016 17 109 15.6 [8.8; 22.4] 14.4%
Ng 2009 35 148 23.6 [16.8; 30.5] 14.4%
Yellapragada 2020 453 1889 24.0 [22.1; 25.9] 14.6%
Pasamonte 2019 7 22 31.8 [12.4; 51.3] 13.2%
Oortgiesen 2019 106 120 88.3 [82.6; 94.1] 14.5%
Yokus 2017 29 31 93.5 [84.9; 100.0] 14.3%
Random effects model 2488 41.6 [19.3; 64.0] 100.0%
Heterogeneity: I^2 = 99%, τ^2 = 0.0888, χ^2_6 = 696.33 (p < 0.01)
0 20 40 60 80 100
Prevalence (%)

D

Study ID	Cases	Total	Prevalence	95% C.I.	Weight
**Vitamin D insufficiency (Under treatment)**					
Yokus 2017	1	31	3.2	[0.0; 9.4]	14.5%
Greenfield 2014	7	32	21.9	[7.6; 36.2]	13.8%
Diamond 2010	24	108	22.2	[14.4; 30.1]	14.4%
Wang 2016	29	109	26.6	[18.3; 34.9]	14.4%
Nath 2019	14	41	34.1	[19.6; 48.7]	13.8%
Oortgiesen 2019	65	120	54.2	[45.3; 63.1]	14.3%
Yellapragada 2020	1190	1889	63.0	[60.8; 65.2]	14.7%
**Random effects model**		**2330**	**32.3**	**[10.0; 54.5]**	**100.0%**

Heterogeneity: $I^2 = 99\%$, $\tau^2 = 0.0876$, $\chi^2_6 = 448.66$ ($p < 0.01$)

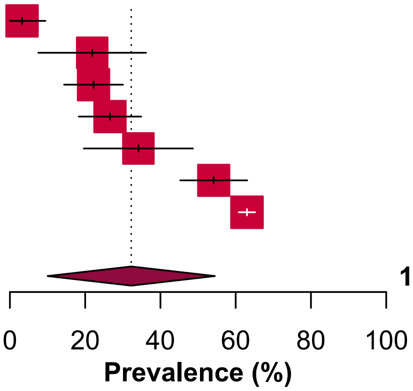

E

Study ID	Cases	Total	Prevalence	95% C.I.	Weight
**Vitamin D deficiency (Europe)**					
Gray 2018	7	88	8.0	[2.3; 13.6]	14.4%
Lauter 2015	27	83	32.5	[22.5; 42.6]	14.3%
Greenfield 2014	12	32	37.5	[20.7; 54.3]	13.9%
Laroche 2010	26	39	66.7	[51.9; 81.5]	14.1%
Oortgiesen 2019	106	120	88.3	[82.6; 94.1]	14.4%
Yokus 2017	29	31	93.5	[84.9; 100.0]	14.4%
Graklanov 2020	36	37	97.3	[92.1; 100.0]	14.5%
**Random effects model**		**430**	**60.7**	**[29.4; 91.9]**	**100.0%**

Heterogeneity: $I^2 = 99\%$, $\tau^2 = 0.1751$, $\chi^2_6 = 693.21$ ($p < 0.01$)

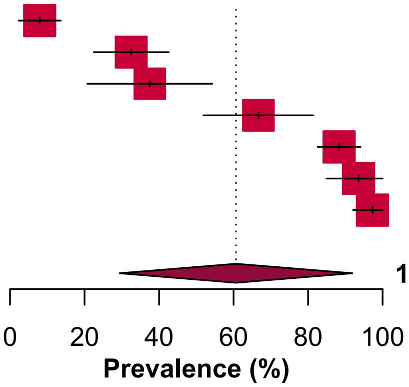

F

Study ID	Cases	Total	Prevalence	95% C.I.	Weight
**Vitamin D insufficiency (Europe)**					
Graklanov 2020	1	37	2.7	[0.0; 7.9]	17.2%
Yokus 2017	1	31	3.2	[0.0; 9.4]	17.1%
Gray 2018	9	88	10.2	[3.9; 16.6]	17.0%
Greenfield 2014	7	32	21.9	[7.6; 36.2]	15.6%
Oortgiesen 2019	65	120	54.2	[45.3; 63.1]	16.7%
Lauter 2015	45	83	54.2	[43.5; 64.9]	16.4%
**Random effects model**		**391**	**24.1**	**[6.4; 41.8]**	**100.0%**

Heterogeneity: $I^2 = 97\%$, $\tau^2 = 0.0469$, $\chi^2_5 = 163.69$ ($p < 0.01$)

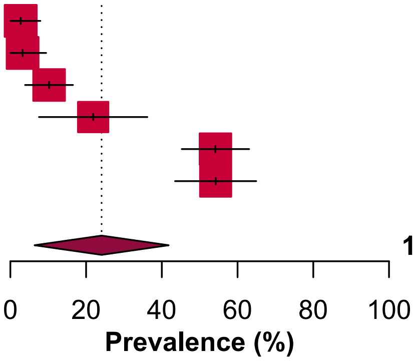

G

Study ID	Cases	Total	Prevalence	95% C.I.	Weight
**Vitamin D deficiency (North America)**					
Hudzik 2015	52	675	7.7	[5.7; 9.7]	17.9%
Ravenborg 2014	24	169	14.2	[8.9; 19.5]	16.9%
Wang 2016	17	109	15.6	[8.8; 22.4]	16.2%
Ng 2009	35	148	23.6	[16.8; 30.5]	16.2%
Yellapragada 2020	453	1889	24.0	[22.1; 25.9]	17.9%
Badros 2008	40	100	40.0	[30.4; 49.6]	14.7%
**Random effects model**		**3090**	**20.4**	**[11.8; 28.9]**	**100.0%**

Heterogeneity: $I^2 = 97\%$, $\tau^2 = 0.0105$, $\chi^2_5 = 159.30$ ($p < 0.01$)

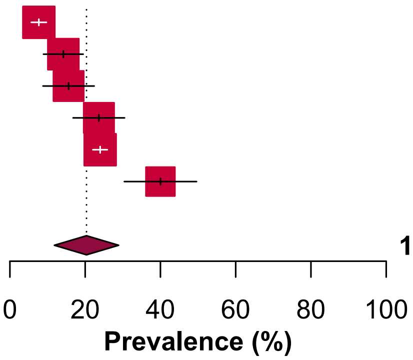

H

Study ID	Cases	Total	Prevalence	95% C.I.	Weight
**Vitamin D insufficiency (North America)**					
Ravenborg 2014	37	169	21.9	[15.7; 28.1]	20.0%
Wang 2016	29	109	26.6	[18.3; 34.9]	19.6%
Badros 2008	35	100	35.0	[25.7; 44.3]	19.3%
Hudzik 2015	394	675	58.4	[54.7; 62.1]	20.5%
Yellapragada 2020	1190	1889	63.0	[60.8; 65.2]	20.6%
**Random effects model**		**2942**	**41.3**	**[25.5; 57.0]**	**100.0%**

Heterogeneity: $I^2 = 98\%$, $\tau^2 = 0.0312$, $\chi^2_4 = 223.68$ ($p < 0.01$)

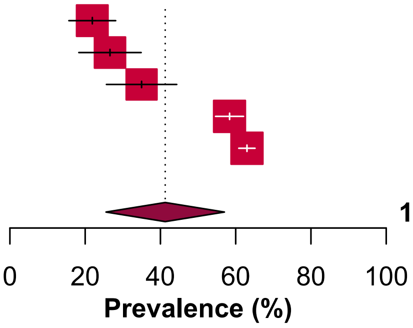

I

Study ID	Cases	Total	Prevalence	95% C.I.	Weight
**Vitamin D deficiency (Australia)**					
Nath 2019	11	41	26.8	[13.3; 40.4]	29.4%
Diamond 2010	34	108	31.5	[22.7; 40.2]	70.6%
**Random effects model**		**149**	**30.1**	**[22.8; 37.5]**	**100.0%**

Heterogeneity: $I^2 = 0\%$, $\tau^2 = 0$, $\chi^2_1 = 0.32$ ($p = 0.57$)

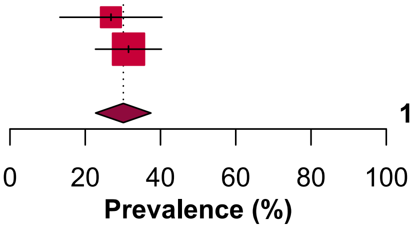

J

Study ID	Cases	Total	Prevalence	95% C.I.	Weight
**Vitamin D insufficiency (Australia)**					
Diamond 2010	24	108	22.2	[14.4; 30.1]	63.7%
Nath 2019	14	41	34.1	[19.6; 48.7]	36.3%
**Random effects model**		**149**	**26.6**	**[15.3; 37.8]**	**100.0%**

Heterogeneity: $I^2 = 50\%$, $\tau^2 = 0.0036$, $\chi^2_1 = 2.01$ ($p = 0.16$)

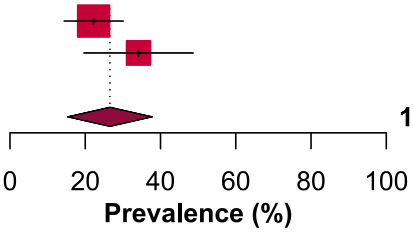

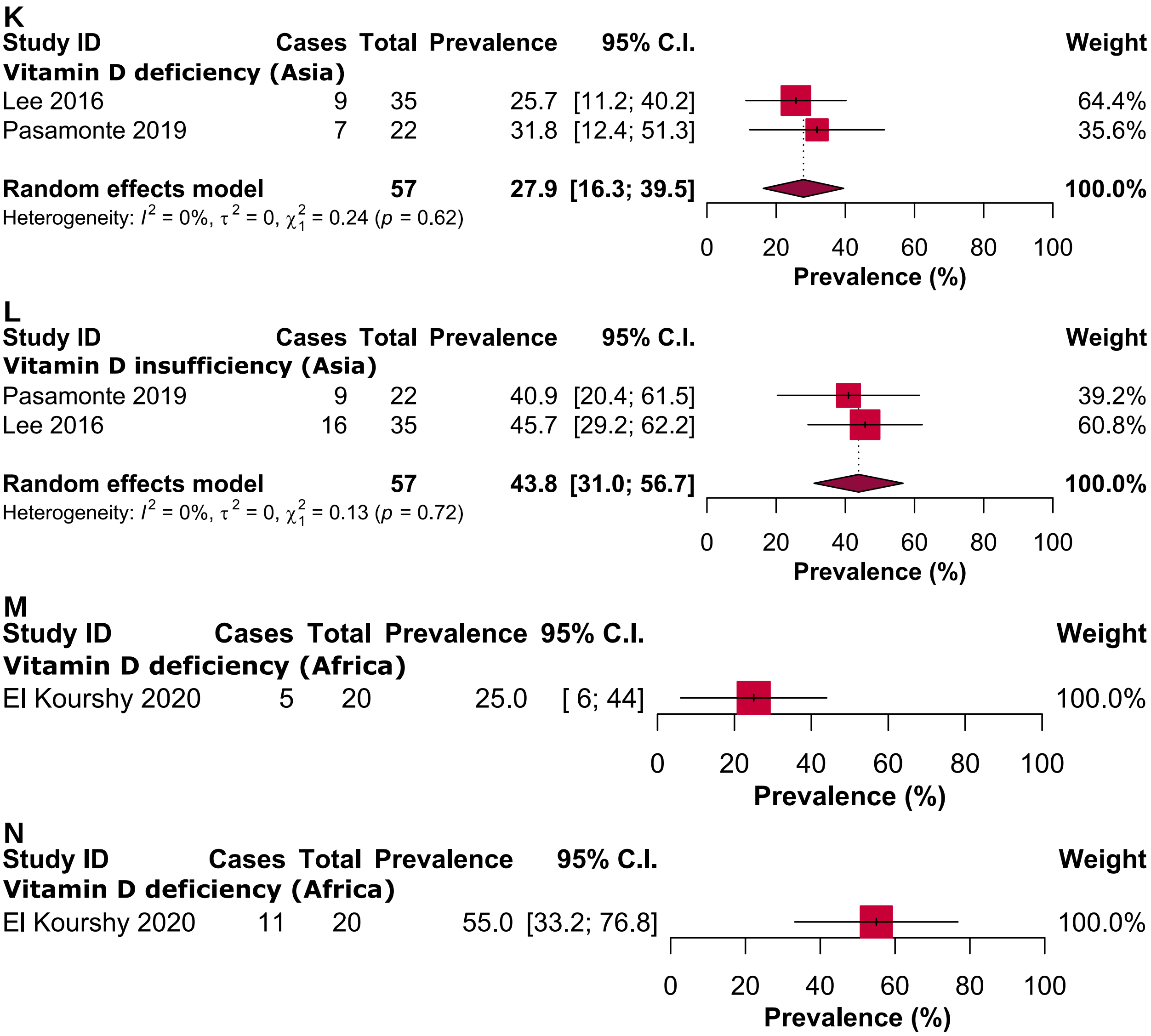

Figure S1. Subgroup analyses estimating the prevalence of vitamin D deficiency and insufficiency in (A-B) newly diagnosed, (C-D) under treatment and multiple myeloma patients from (E-N) different regions.

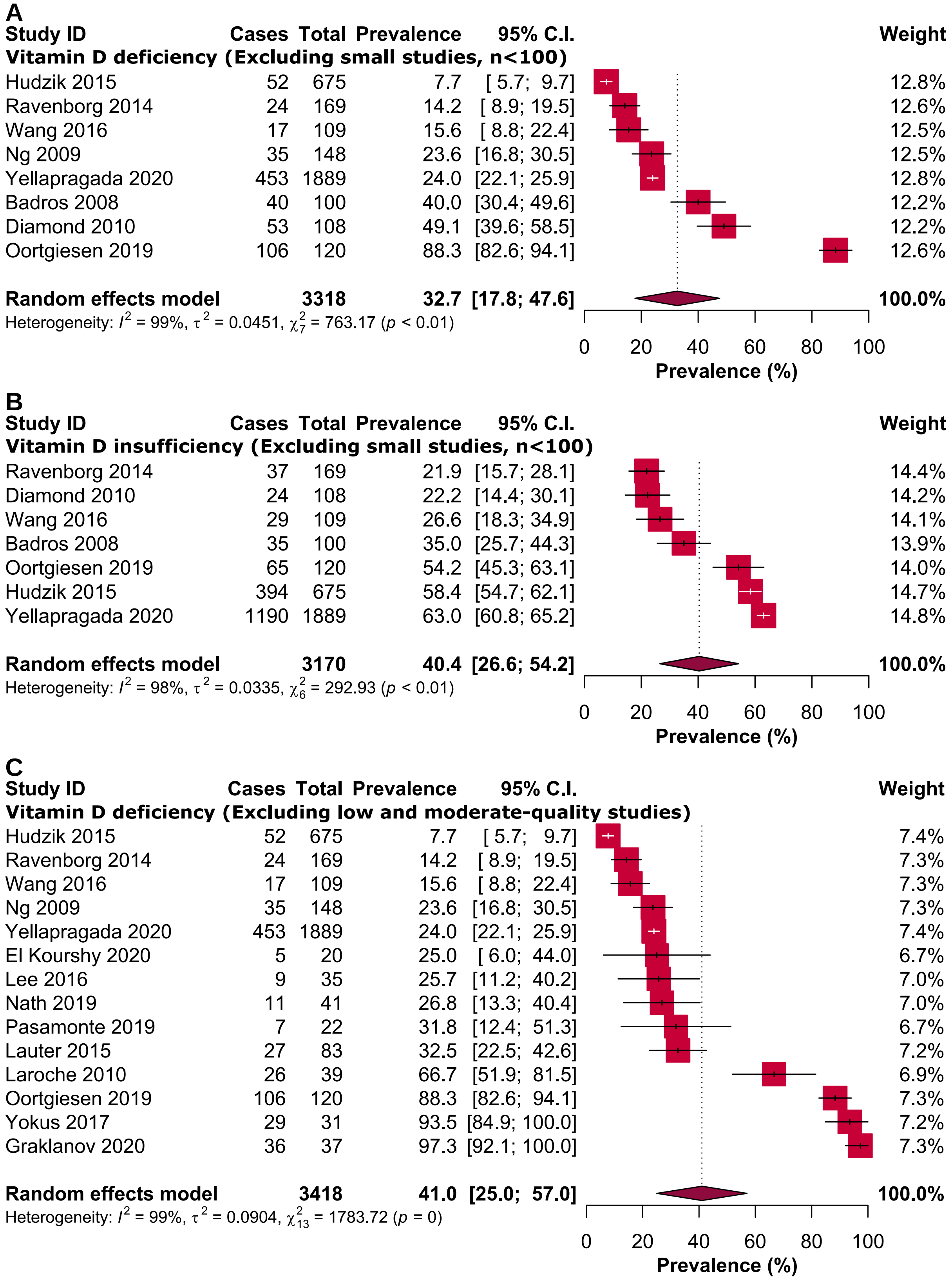
A
Study ID Cases Total Prevalence 95% C.I. Weight
Vitamin D deficiency (Excluding small studies, n<100)
Hudzik 2015 52 675 7.7 [5.7; 9.7] 12.8%
Ravenborg 2014 24 169 14.2 [8.9; 19.5] 12.6%
Wang 2016 17 109 15.6 [8.8; 22.4] 12.5%
Ng 2009 35 148 23.6 [16.8; 30.5] 12.5%
Yellapragada 2020 453 1889 24.0 [22.1; 25.9] 12.8%
Badros 2008 40 100 40.0 [30.4; 49.6] 12.2%
Diamond 2010 53 108 49.1 [39.6; 58.5] 12.2%
Oortgiesen 2019 106 120 88.3 [82.6; 94.1] 12.6%
Random effects model 3318 32.7 [17.8; 47.6] 100.0%
Heterogeneity: I^2 = 99%, τ^2 = 0.0451, χ^2_7 = 763.17 (p < 0.01)
0 20 40 60 80 100
Prevalence (%)
B
Study ID Cases Total Prevalence 95% C.I. Weight
Vitamin D insufficiency (Excluding small studies, n<100)
Ravenborg 2014 37 169 21.9 [15.7; 28.1] 14.4%
Diamond 2010 24 108 22.2 [14.4; 30.1] 14.2%
Wang 2016 29 109 26.6 [18.3; 34.9] 14.1%
Badros 2008 35 100 35.0 [25.7; 44.3] 13.9%
Oortgiesen 2019 65 120 54.2 [45.3; 63.1] 14.0%
Hudzik 2015 394 675 58.4 [54.7; 62.1] 14.7%
Yellapragada 2020 1190 1889 63.0 [60.8; 65.2] 14.8%
Random effects model 3170 40.4 [26.6; 54.2] 100.0%
Heterogeneity: I^2 = 98%, τ^2 = 0.0335, χ^2_6 = 292.93 (p < 0.01)
0 20 40 60 80 100
Prevalence (%)
C
Study ID Cases Total Prevalence 95% C.I. Weight
Vitamin D deficiency (Excluding low and moderate-quality studies)
Hudzik 2015 52 675 7.7 [5.7; 9.7] 7.4%
Ravenborg 2014 24 169 14.2 [8.9; 19.5] 7.3%
Wang 2016 17 109 15.6 [8.8; 22.4] 7.3%
Ng 2009 35 148 23.6 [16.8; 30.5] 7.3%
Yellapragada 2020 453 1889 24.0 [22.1; 25.9] 7.4%
El Kourshy 2020 5 20 25.0 [6.0; 44.0] 6.7%
Lee 2016 9 35 25.7 [11.2; 40.2] 7.0%
Nath 2019 11 41 26.8 [13.3; 40.4] 7.0%
Pasamonte 2019 7 22 31.8 [12.4; 51.3] 6.7%
Lauter 2015 27 83 32.5 [22.5; 42.6] 7.2%
Laroche 2010 26 39 66.7 [51.9; 81.5] 6.9%
Oortgiesen 2019 106 120 88.3 [82.6; 94.1] 7.3%
Yokus 2017 29 31 93.5 [84.9; 100.0] 7.2%
Graklanov 2020 36 37 97.3 [92.1; 100.0] 7.3%
Random effects model 3418 41.0 [25.0; 57.0] 100.0%
Heterogeneity: I^2 = 99%, τ^2 = 0.0904, χ^2_{13} = 1783.72 (p = 0)
0 20 40 60 80 100
Prevalence (%)

D

Study ID	Cases	Total	Prevalence	95% C.I.	Weight
**Vitamin D insufficiency (Excluding low and moderate-quality studies)**					
Graklanov 2020	1	37	2.7	[0.0; 7.9]	8.7%
Yokus 2017	1	31	3.2	[0.0; 9.4]	8.6%
Ravenborg 2014	37	169	21.9	[15.7; 28.1]	8.6%
Wang 2016	29	109	26.6	[18.3; 34.9]	8.5%
Nath 2019	14	41	34.1	[19.6; 48.7]	8.1%
Pasamonte 2019	9	22	40.9	[20.4; 61.5]	7.6%
Lee 2016	16	35	45.7	[29.2; 62.2]	8.0%
Oortgiesen 2019	65	120	54.2	[45.3; 63.1]	8.5%
Lauter 2015	45	83	54.2	[43.5; 64.9]	8.4%
El Kourshy 2020	11	20	55.0	[33.2; 76.8]	7.4%
Hudzik 2015	394	675	58.4	[54.7; 62.1]	8.7%
Yellapragada 2020	1190	1889	63.0	[60.8; 65.2]	8.8%
**Random effects model**		**3231**	**38.1**	**[22.7; 53.4]**	**100.0%**

Heterogeneity: $I^2 = 99\%$, $\tau^2 = 0.0698$, $\chi^2_{11} = 800.09$ ($p < 0.01$)

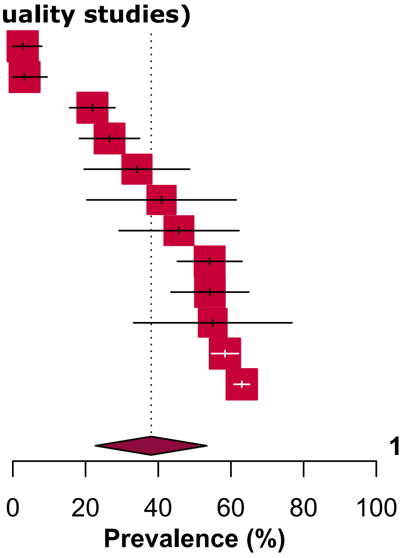

E

Study ID	Cases	Total	Prevalence	95% C.I.	Weight
**Vitamin D deficiency (Considering only cross-sectional studies)**					
Hudzik 2015	52	675	7.7	[5.7; 9.7]	14.5%
Lee 2016	9	35	25.7	[11.2; 40.2]	14.2%
Nath 2019	11	41	26.8	[13.3; 40.4]	14.3%
Pasamonte 2019	7	22	31.8	[12.4; 51.3]	14.0%
Greenfield 2014	12	32	37.5	[20.7; 54.3]	14.1%
Oortgiesen 2019	106	120	88.3	[82.6; 94.1]	14.5%
Graklanov 2020	36	37	97.3	[92.1; 100.0]	14.5%
**Random effects model**		**962**	**45.2**	**[5.8; 84.5]**	**100.0%**

Heterogeneity: $I^2 = 100\%$, $\tau^2 = 0.2783$, $\chi^2_6 = 1487.61$ ($p < 0.01$)

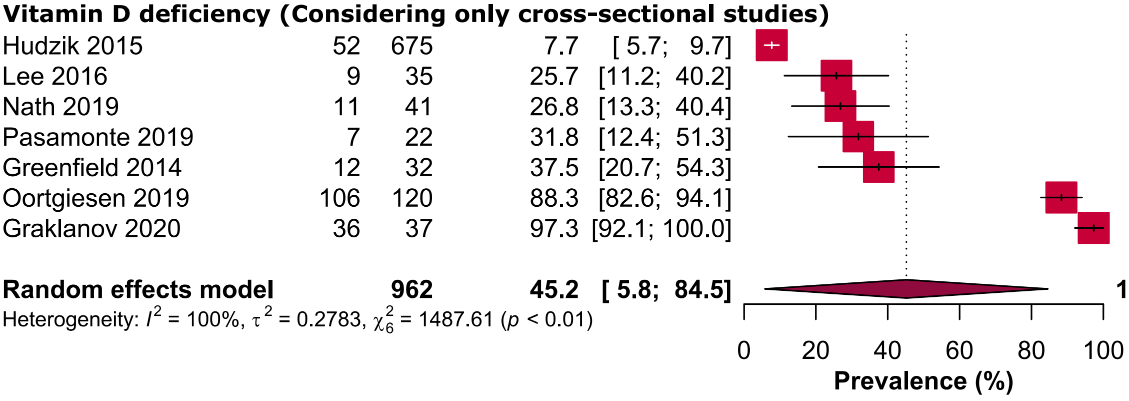

F

Study ID	Cases	Total	Prevalence	95% C.I.	Weight
**Vitamin D insufficiency (Considering only cross-sectional studies)**					
Graklanov 2020	1	37	2.7	[0.0; 7.9]	17.3%
Greenfield 2014	7	32	21.9	[7.6; 36.2]	16.6%
Nath 2019	14	41	34.1	[19.6; 48.7]	16.6%
Pasamonte 2019	9	22	40.9	[20.4; 61.5]	15.8%
Lee 2016	16	35	45.7	[29.2; 62.2]	16.3%
Hudzik 2015	394	675	58.4	[54.7; 62.1]	17.4%
**Random effects model**		**842**	**33.8**	**[7.2; 60.5]**	**100.0%**

Heterogeneity: $I^2 = 98\%$, $\tau^2 = 0.1063$, $\chi^2_5 = 296.19$ ($p < 0.01$)

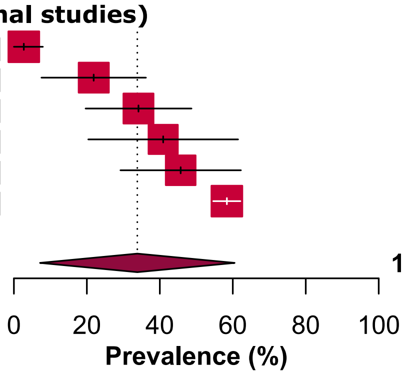

Figure S2. Sensitivity analyses (A-B) excluding small studies, (C-D) excluding low- and moderate-quality studies and (E-F) considering only cross-sectional studies estimating the prevalence of vitamin D deficiency and insufficiency in multiple myeloma patients.

Table S1. Search strategies

Databases	Search strategies
**PubMed**	(myeloma[Title/Abstract] OR “plasma cell dyscrasias”[Title/Abstract] OR myelomatosis[Title/Abstract] OR myelomatoses[Title/Abstract] OR “Kahler's disease”[Title/Abstract] OR “Kahler disease”[Title/Abstract]) AND ("vitamin D"[Title/Abstract] OR hypovitaminosis[Title/Abstract] OR hydroxyvitamin[Title/Abstract] OR "25 OH D"[Title/Abstract])
**Scopus**	TITLE-ABS(myeloma OR "plasma cell dyscrasias" OR myelomatosis OR myelomatoses OR "Kahler's disease" OR "Kahler disease") AND TITLE-ABS("vitamin D" OR "hypovitaminosis" OR hydroxyvitamin OR "25 OH D")
**Web of Science**	#1<br>TI=(myeloma OR “plasma cell dyscrasias” OR myelomatosis OR myelomatoses OR “Kahler's disease” OR “Kahler disease”) AND TI=("vitamin D" OR "hypovitaminosis" OR hydroxyvitamin OR "25 OH D")<br>Indexes=SCI-EXPANDED, SSCI, A&HCI, CPCI-S, CPCI-SSH, BKCI-S, BKCI-SSH, ESCI Timespan=All years<br>#2<br>AB=(myeloma OR “plasma cell dyscrasias” OR myelomatosis OR myelomatoses OR “Kahler's disease” OR “Kahler disease”) AND AB=("vitamin D" OR "hypovitaminosis" OR hydroxyvitamin OR "25 OH D")<br>Indexes=SCI-EXPANDED, SSCI, A&HCI, CPCI-S, CPCI-SSH, BKCI-S, BKCI-SSH, ESCI Timespan=All years<br>#1 OR #2<br>Indexes=SCI-EXPANDED, SSCI, A&HCI, CPCI-S, CPCI-SSH, BKCI-S, BKCI-SSH, ESCI Timespan=All years
**Google Scholar**	allintitle:(myeloma OR “plasma cell dyscrasias” OR myelomatosis OR myelomatoses OR “Kahler's disease” OR “Kahler disease”) ("vitamin D" OR "hypovitaminosis" OR hydroxyvitamin OR "25 OH D")

**ScienceDirect**	Title, abstract, keywords: (myeloma OR “plasma cell dyscrasias” OR myelomatosis OR myelomatoses OR “Kahler's disease” OR “Kahler disease”) ("vitamin D" OR "hypovitaminosis" OR hydroxyvitamin OR "25 OH D")

Table S2. Quality assessment of the included cohort studies

No.	Study ID	Questions assessing the included cohort studies											Yes (%)
		1	2	3	4	5	6	7	8	9	10	11	
1	Badros 2008	U	Y	Y	Y	U	U	U	Y	NA	NA	Y	55.5
2	Diamond 2010	U	Y	Y	Y	Y	Y	Y	U	U	U	Y	63.6
3	Gray 2018	U	Y	U	Y	N	Y	U	Y	N	N	N	36.4
4	Laroche 2010	Y	Y	Y	U	U	Y	Y	Y	Y	NA	Y	80.0
5	Lauter 2015	Y	Y	Y	Y	U	Y	Y	Y	Y	Y	Y	90.9
6	Ng 2009	Y	Y	Y	Y	U	U	Y	Y	NA	NA	Y	77.7
7	Ravenborg 2014	Y	Y	Y	U	U	Y	Y	Y	Y	NA	Y	80.0
8	Wang 2016	Y	Y	Y	U	U	Y	Y	Y	NA	NA	Y	77.7
9	Yellapragada 2020	Y	Y	Y	U	U	Y	Y	Y	U	U	Y	63.6
10	Yokus 2017	Y	Y	Y	Y	Y	Y	Y	Y	Y	Y	Y	100.0

1. Were the two groups similar and recruited from the same population? 2. Were the exposures measured similarly to assign people to both exposed and unexposed groups? 3. Was the exposure measured in a valid and reliable way? 4. Were confounding factors identified? 5. Were strategies to deal with confounding factors stated? 6. Were the groups/participants free of the outcome at the start of the study (or at the moment of exposure)? 7. Were the outcomes measured in a valid and reliable way? 8. Was the follow up time reported and sufficient to be long enough for outcomes to occur? 9. Was follow up complete, and if not, were the reasons to loss to follow up described and explored? 10. Were strategies to address incomplete follow up utilized? 11. Was appropriate statistical analysis used? Y=Yes; N=No; U=Unclear, NA=Not applicable.

Table S3. Quality assessment of the included cross-sectional studies

No.	Study ID	Questions assessing the included cross-sectional studies								Yes (%)
		1	2	3	4	5	6	7	8	
1	Graklanov 2020	Y	Y	Y	Y	U	U	Y	Y	75.0
2	Greenfield 2014	Y	Y	Y	Y	U	U	Y	N	62.5
3	Hudzik 2015	Y	Y	Y	Y	U	U	Y	Y	75.0
4	Lee 2016	Y	Y	Y	Y	U	U	Y	Y	75.0
5	Nath 2019	Y	Y	Y	Y	Y	Y	Y	Y	100.0
6	Oortgiesen 2019	Y	Y	Y	Y	U	U	Y	Y	75.0
7	Pasamonte 2019	Y	Y	Y	Y	U	U	Y	Y	75.0

1. Were the criteria for inclusion in the sample clearly defined? 2. Were the study subjects and the setting described in detail? 3. Was the exposure measured in a valid and reliable way? 4. Were objective, standard criteria used for measurement of the condition? 5. Were confounding factors identified? 6. Were strategies to deal with confounding factors stated? 7. Were the outcomes measured in a valid and reliable way? 8. Was appropriate statistical analysis used?

Table S4. Quality assessment of the included case-control studies

No.	Study ID	Questions assessing the included case-control studies										Yes (%)
		1	2	3	4	5	6	7	8	9	10	
1	El Koursh 2020	Y	Y	Y	Y	Y	N	N	Y	Y	Y	80.0

1. Were the groups comparable other than the presence of disease in cases or the absence of disease in controls? 2. Were cases and controls matched appropriately? 3. Were the same criteria used for identification of cases and controls? 4. Was exposure measured in a standard, valid and reliable way? 5. Was exposure measured in the same way for cases and controls? 6. Were confounding factors identified? 7. Were strategies to deal with confounding factors stated? 8. Were outcomes assessed in a standard, valid and reliable way for cases and controls? 9. Was the exposure period of interest long enough to be meaningful? 10. Was appropriate statistical analysis used? Y=Yes; N=No; U=Unclear.
